# *Cryptococcus neoformans* osteomyelitis of the tibia: a case report and review of the literature

**DOI:** 10.1186/s13256-023-03925-x

**Published:** 2023-05-07

**Authors:** Stamatios A. Papadakis, Georgios Gourtzelidis, Dimitrios Pallis, Margarita-Michaela Ampadiotaki, Fotios Tatakis, Konstantinos Tsivelekas, Kleoniki Georgousi, Constantinos Kokkinis, Kalliopi Diamantopoulou, Moyssis Lelekis

**Affiliations:** 1 B’ Department of Orthopaedics, KAT General Hospital of Attica, 2 Nikis Street, 14561 Kifisia, Greece; 2 Department of Internal Medicine, KAT General Hospital of Attica, Kifisia, Greece; 3 Department of Radiology, KAT General Hospital of Attica, Kifisia, Greece; 4 Department of Pathology, KAT General Hospital of Attica, Kifisia, Greece

**Keywords:** *Cryptococcus*, Osteomyelitis, Tibia, Breast, Case report

## Abstract

**Introduction:**

Osteomyelitis is a bone inflammation that can be related to various infectious agents. As with any other type of inflammation, the prevailing symptoms and signs may include redness, swelling, pain, and heat. Fungal osteomyelitis is rare and usually found in immune-compromised patients.

**Case presentation:**

A non-human immunodeficiency virus immunocompromised Greek female patient, 82 years old, visited the emergency department due to a 3 day pain located mainly over the left tibia’s anterior surface, accompanied by swelling and redness. There was also a subcutaneous lesion of her left breast. Medical history revealed that the patient had an unmasked close contact with pigeons, a main host of the disease. Initial x-ray imaging showed an osteolytic area in the upper third of the tibial diaphysis. The patient was admitted and underwent a computed tomography-guided biopsy. The specimen revealed a *Cryptococcus neoformans* infection of the bone and the breast. She was treated with 400 mg fluconazole twice a day for 3 weeks while in hospital and 200 mg twice a day upon discharge for 9 months. After that, she underwent surgical debridement because of lasting local irritation. She was closely monitored in our outpatient office, and in her last visit, 1 year after the initial admission, inflammatory signs had regressed vastly.

**Conclusions:**

To our knowledge, this is the ninth cryptococcal osteomyelitis of the tibia to be recorded since 1974, and the most unusual finding was the bifocal nature of the infection, affecting both the tibia and the breast.

## Background

The most common type of pathogen in orthopedic patients with osteomyelitis is bacteria, yet a rarer and worth mentioning category are the different fungi. In that case, the clinical entity is described as fungal osteomyelitis. Some usual suspects include the various species of *Candida*, *Cryptococcus*, *Aspergillus*, and so on. *Cryptococcus*, a haploid budding yeast [1], was first isolated from the environment, and the first case of human transmission was osteomyelitis of the tibia [2]. It is an encapsulated fungus falling in the yeast category, and its infection typically causes symptoms from the pulmonary and central nervous systems in human immunodeficiency virus (HIV) patients. The typical scenario starts with the patient inhaling *Cryptococcus*, which is then transmitted through the blood circulation to various organs and sites in the body, with a particular preference for the central nervous system [3]. *Cryptococcus neoformans* is the most prevalent form worldwide. It is present in the soil, avian excreta (mainly pigeons), and in some specific types of trees. It is typically associated with impaired cellular immunity. It is believed to enter the host’s body via the respiratory system and then migrate, usually into the brain [4].

Cryptococcosis is an opportunistic infection in patients from areas endemic for HIV. Though extremely rare, cryptococcal osteomyelitis could also occur in an otherwise healthy immune-competent individual [5] and coexist with other clinical entities, such as sarcoidosis and tuberculosis [6, 7]. It may or may not be accompanied by periosteal reaction [1]. Defective cell immunity seems to play a vital role [8]. A cryptococcal lesion could prepare the ground for a pathologic fracture in a way that disturbs the distinctive properties of the bone. The periosteal reaction may be present in conventional radiography, and bone scintigraphy reveals increased activity [5]. Inflammation indicators such as erythrocyte sedimentation rate, white blood cell count, and C-reactive protein may be increased. If left untreated, cryptococcosis could result in chronic osteomyelitis. Thus, a fracture that follows the pathologic pattern could be the first sign of the disease [5]. After initial fixation of such a fracture and surgical debridement, the gold standard for pharmacological treatment is either a regimen with amphotericin B (1 mg/kg/day) or fluconazole 400 mg daily, with stepping down to 200 mg after 2–3 months for a total timeline of 6 months. Some authors suggest a combination of the drugs, as mentioned above, through the initial steps of treatment.

## Case

An 82-year-old Greek female presented in the emergency department with a history of a 3 day pain located mainly over the left anterior upper tibia, accompanied by swelling and redness (day 0). She was a diabetic patient with arterial hypertension, chronic kidney disease, hypothyroidism, and rheumatoid arthritis under therapy with methotrexate (2.5 mg twice per week) and corticosteroids (4 mg of methylprednisolone twice a day). The patient was afebrile. The physical examination revealed a well-circumscribed ulcer on the upper tibia, approximately 3 cm in diameter. Simultaneously, she had a second well-circumscribed ulcerative lesion over the upper out quarter of her left breast. Her laboratory results were as follows: white blood cells, 5.63 × 10^3^/μL with 4.09 × 10^3^/μL neutrophils and 1.02 × 10^3^/μL lymphocytes; hemoglobin, 10.1 g/dL; platelets, 280 × 10^3^/μL, and serum creatinine, 1.31 mg/dL. Serology testing for HIV was negative. The inflammatory markers were as follows: C reactive protein (CRP) was 2.99 mg/dL (normal range 0–0.5 mg/dL), erythrocyte sedimentation rate (ESR) was 81 mm/hour (normal range 0–20 mm/hour), and procalcitonin (PCT) was 0.13 ng/mL (normal range < 0.5 ng/mL).

**Fig. 1:**
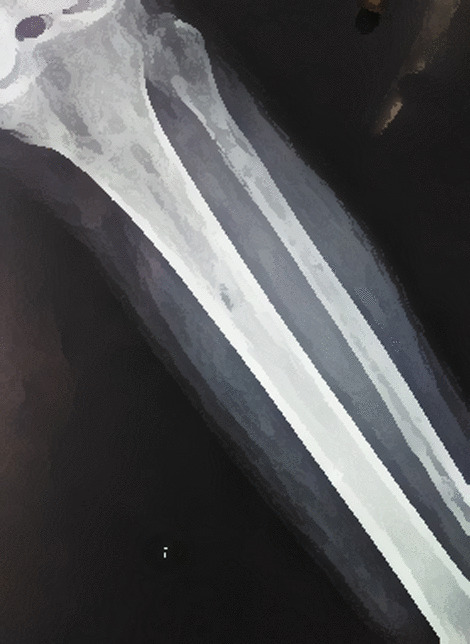
Anteroposterior x-ray of the patient’s left tibia and the fibula. Initial x-rays upon arrival, indicating an osteolytic lesion of the diaphysis

**Fig. 2:**
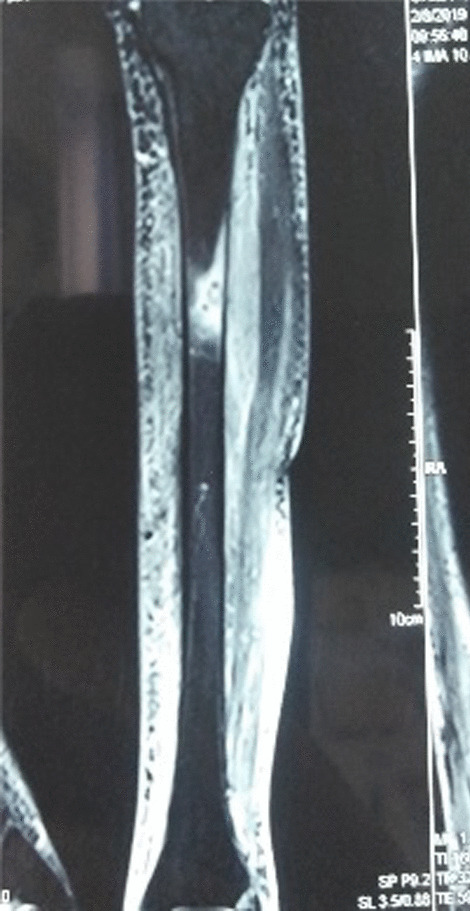
MRI axial view of the affected tibia, STIR sequence showing a high-signal, osteolytic-like lesion of the diaphysis

**Fig. 3:**
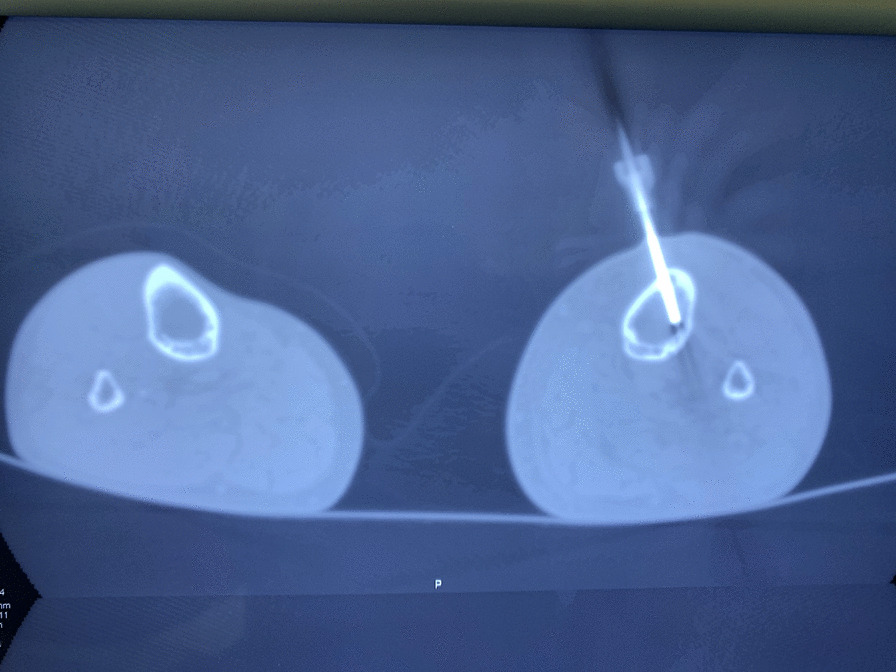
CT-guided biopsy of the left tibia

**Fig. 4:**
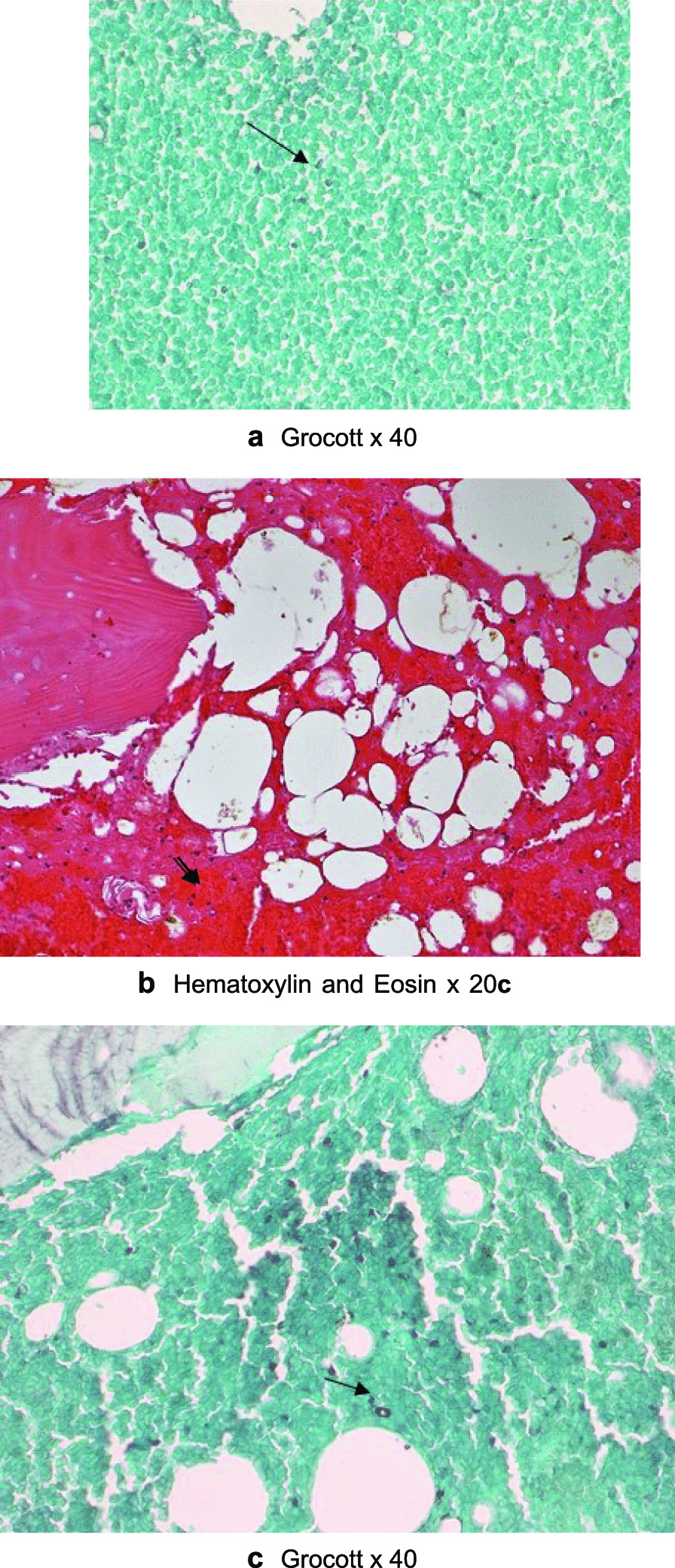
**a**–**c** Microscopic images of the bone and marrow specimen depicting the fungi in arrows

The patient’s x-ray revealed an osteolytic area in the upper third of the tibial diaphysis (Fig. 1). Magnetic resonance imaging (ΜRI) on day 3 revealed a lesion of the bone marrow in the upper part of the diaphysis, with a heterogeneous pathological composition approximately 3 cm long (Fig. 2). It also described edema of the soft tissue. Furthermore, there was a minor injury of the nearby bone cortex and the periosteum. The patient underwent a computed tomography (CT)-guided biopsy at the lesion (day 6, Fig. 3) over the anterior surface of the tibia. The specimen was cultivated and revealed a *Cryptococcus neoformans* infection of the bone (Fig. 4). The brain and chest CT scan at day 15 did not reveal pathological findings.

**Fig. 5:**
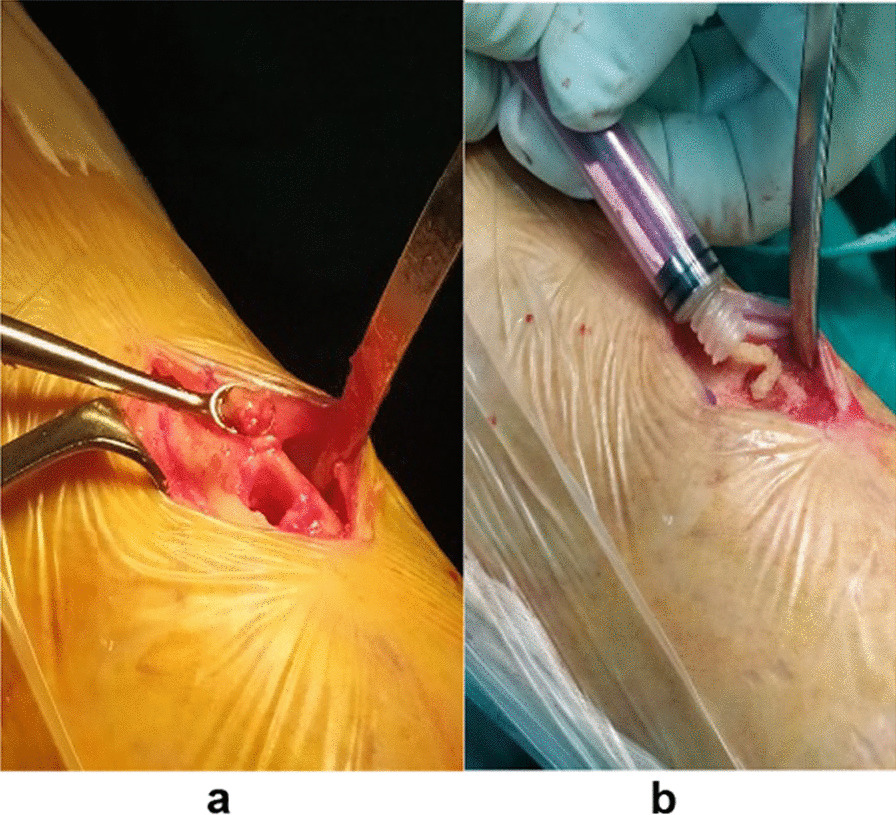
Intraoperative images. **a** Debridement of the lesion. **b** Filling with bony allograft and 50 mg of amphotericin B powder

Moreover, the patient underwent an excisional biopsy at the second lesion over her left breast, which was also positive for *C. neoformans*. The identification was based on colonial morphology, a positive India ink test, and the ID32C API system. Lumbar puncture and blood cultures were negative (day 20). Since the *C. neoformans* strain was susceptible to flucytosine, posaconazole, voriconazole, itraconazole, fluconazole, and amphotericin B, treatment with fluconazole was decided (loading dose of 800 mg and maintenance dose of 400 mg/day intravenous) for 3 weeks initially. The patient was treated with 200 mg fluconazole twice a day for 9 months total duration. She did not undergo surgical debridement initially since she responded well to antifungal pharmacotherapy alone at first [9]. However, 9 months after the treatment initiation, some minor regional discomfort persisted over the affected area in tibia. No discomfort was noted over the previously affected breast. The decision was made for the patient to undergo surgical debridement to eliminate the infection of the bone while still receiving the same antifungal regimen per os. With an anterior tibial direct approach at day 289, the affected area was recognized, and the infected bone was marginally removed under natural vision. The bone marrow was washed with 50 mg amphotericin B solution, and the bony gap was covered with 2.5 mL bony allograft and 50 mg of amphotericin B powder (Fig. 5).

**Fig. 6:**
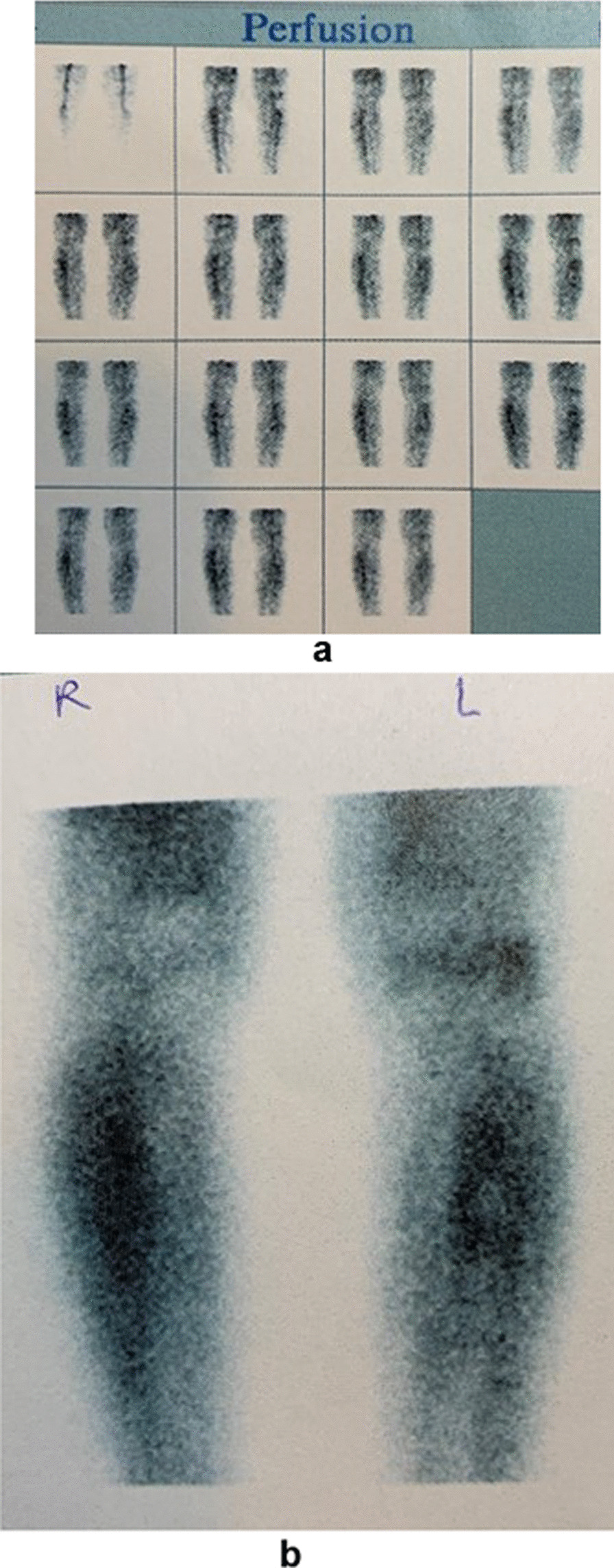
**a**, **b** Images depicting the bone scintigraphy of both patient’s tibiae with the same perfusion and signal

The patient was not adherent to the standard antithrombotic regimen prescribed. As a result, she developed a deep vein thrombosis of the affected leg 1 month after the surgical intervention. She was hospitalized for a week in the vascular surgery department and was discharged with no further complications. Her wound was healed adequately by that time and showed no signs of local inflammation. She was discharged from the hospital, continuing the oral antifungal regimen (fluconazole as previously administered) for another 3 months, when she had her subsequent CT-guided needle biopsy, which proved negative for the fungus. As the last confirmation, the patient underwent a bone scintigraphy 12 months after the initial hospitalization, which also proved negative for inflammation (Fig. 6). No persisting symptoms were referred by the patient at this point and the patient was declared free of the disease.

## Review of the literature

Apropos of this case report, a review in the English literature from 1974 to 2022 was performed, as Nottebart *et al*. [10] published a review paper in 1974, and described 37 cases confirmed by histology or culture of *C. neoformans*. The authors stated that bony involvement due to *Cryptococcus* species is uncommon, with an estimated prevalence of 5%, and virtually every bone could be involved. The current review revealed 106 more cases in adults including this present report [4–8, 11–82] (Table 1).

**Table 1:** Published cases of cryptococcal osteomyelitis based on frequency of the affected site since 1974

Location	No. cases	References
Vertebrae	21	[7, 12–14, 33, 37–40(7), 41–45, 65]
Rib	18	[15, 16(2), 26–36, 72, 75, 81, 82]
Femur	11	[11, 22, 31, 33, 52–56, 69, 73]
Skull	10	[11, 19, 20, 46–51, 66]
Humerus	10	[5, 6, 11, 18, 23, 46, 57, 58, 71, 78]
Tibia	9	[6, 8, 17, 24, 68, (ps)]
Scapula	3	[11, 15, 17]
Clavicle	3	[58, 62, 67]
Sacrum	3	[33, 39, 43]
Ilium + pubis	3	[59, 60, 80]
Mandible + zygomatic	2	[36, 51]
Ulna	2	[11, 79]
Phalanx	2	[4, 63]
Sternum	1	[21]
Radius	1	[74]
Hand	1	[24]
Metacarpal	1	[64]
Knee joint	1	[70]
Ankle	1	[78]
Talus	1	[76]
Calcaneus	1	[77]
Metatarsal	1	[60]

(ps): Present case; number in brackets: number of more than one cases presented by the same author

The vast majority of these cases involved only one single site, and only 13 cases presented with multiple skeletal lesions [6, 15, 17, 31, 33, 36, 37, 39, 44–46, 58, 78]. Cases that reported cryptococcal osteomyelitis in pediatric patients were excluded.

Immune-compromised patients bear a higher risk of developing the cryptococcal disease, and osteomyelitis due to *C. neoformans* has no age predilection [11]. Isolated fungal osteomyelitis is a rare condition, usually affecting a sole vertebra [12]. As Li *et al*. suggested [13], spinal involvement requires both MRI and laboratory tests to better assess both the pathogen and the lesion’s extent and nature. Surgical treatment should be considered in cases where spinal stability could be compromised [12–14]. Osseous involvement is as a sign of systemic dissemination in most cases. Specific fungal cultures and stains are needed to establish the diagnosis, which is confirmed through a biopsy. *C. neoformans* produces a polysaccharide capsule, which acts as an antigen and can be detected in the blood. It is a valuable biomarker, though its relatively low specificity and sensitivity should be considered before making or excluding a diagnosis [15]. Recently, novel diagnostic tools have been proposed, such as metagenomic next-generation sequencing, for diagnosing cryptococcosis of the rib [16]. The possibility of cryptococcal osteomyelitis in a patient with an osteolytic bone lesion on radiological images should always be kept in mind [17].

The disease appears to be even more infrequent in children, though 17 cases have been recorded [2, 83, 84]. As with adults, HIV-positive children are more prone to suffering from such a condition. The suggested treatment includes a regimen of initial intravenous and later oral fluconazole for 12 weeks, for a minimum of 6 months and even up to 1 year.

Although extremely rare, cryptococcal osteomyelitis should be considered a diagnosis in an immunocompetent patient with a lytic lesion on radiologic studies [15]. It is estimated that 10–40% of patients with cryptococcosis bear no other immune-compromising disease [85, 86]. Possible sites of bony involvement include the femur, humerus [18], tibia, ribs [16], scapula, and most frequent of all, the vertebrae (Table 1). In rare cases involving the skull, a palpable tender mass may be the first sign [19], together or without headache and vomiting [20]. The majority of cases include a single osseous involvement at a time [6]. There is no consensus yet as to how long pharmacotherapy should last. The gold standard regimen for disseminated cryptococcosis recommended by the Infectious Diseases Society of America is a combination of amphotericin B and flucytosine initially, followed by fluconazole up until the end of the treatment. For an immunocompetent patient with a single site involvement, fluconazole monotherapy may suffice [15].

Unusual sites of involvement, such as the sternum, should not be excluded in immunocompetent patients [21]. Early detection and treatment are beneficial for many patients, immunocompromised or not, thus lowering this particular clinical entity’s morbidity and mortality rates.

Patients that have undergone solid organ transplantation (for example, liver) are particularly prone to this kind of infection, and they are also more susceptible to its various complications, even death [22]. Due to the expected drug-induced partial suppression of the immune system, a possible *Cryptococcus* insult is easier to penetrate the circulation, thus acquiring its disseminated systemic form and even local spread into the surrounding muscle and soft tissue.

There are cases of patients whose radiologic findings bear significant resemblance to other known and more common entities such as neoplasms (enchondroma, giant cell tumor) or bacterial infections [23]. In these cases, the patient frequently receives an irrelevant initial treatment before the final and accurate diagnosis is established (for example, antimycobacterial chemotherapy). Surgical debridement and curettage play a vital role in the process of differentiation. Some cases still require additional measures (for example, stabilization with an appropriate site external fixator) to prevent complications postoperatively, such as a pathologic fracture [4].

## Discussion

To our best knowledge, the present case was the fifth cryptococcal osteomyelitis of the tibia to be recorded since 1974. Chokevittaya *et al*. [24] in 2022 published a series of four cases of *C. neoformans* tibial osteomyelitis in Thailand, raising the total number of tibia involvement to nine (Table 2).

**Table 2:** Published cases of tibial cryptococcosis since 1974 in ascending chronological order

References	Comorbidity	Skeletal sites	Treatment
Liu *et al*. [6]	Hepatitis B	Humerus, tibia	Surgical debridement + antifungal treatment
Delat and Laheri [17]	Immunocompetent	Scapula, tibia	Surgical debridement + antifungal treatment
Harirchian *et al*. [8]	Multiple sclerosis	Tibia	Surgical debridement + antifungal treatment
Annapureddy *et al*. [68]	Pregnancy	Tibia	Surgical debridement + antifungal treatment
Chokevittaya *et al*. (4) [24]	NR	Tibia	NR
Present case	Rheumatoid arthritis	Tibia, breast	Surgical debridement + antifungal treatment

Number in brackets: number of more than one cases presented by the same author

*NR* not reported

Although our patient was under treatment with methotrexate due to rheumatoid arthritis, there were no prior complaints from the central nervous or respiratory system, nor a history of trauma. The most unusual finding was the bifocal nature of the infection, affecting both the tibia and the breast. Based on the clinical, biochemical, and radiological findings, she was diagnosed with stage III Bl chronic according to the Cierny–Mader classification [25].

Some specific medical history characteristics may also play an important role, such as recent exposure to pigeons, known carriers of this pathogen. Soft tissue swelling and tenderness are typical cryptococcal infection symptoms, whose clinical and radiological appearance may resemble tuberculosis at first, especially in the spine. Early biopsy and histological examination of the tissue would set the diagnosis and prevent any further dissemination of the disease [14], and this is also what happened in our case.

As with most cases in the literature [9], the patient was initially treated with a standard intravenous antifungal treatment (fluconazole and amphotericin B) that was continued per os for a total of 9 months. Although most cases of cryptococcal osteomyelitis of the tibia are treated surgically (Table 2), the initial plan was to avoid surgery for this patient, due to her personal preferences. As the symptoms persisted, the initial decision for a conservative treatment was revised and the patient underwent surgery. When taking into consideration the consequent relief of the symptoms and the imaging signs, this was the treatment of choice for a patient with this background.

## Conclusion

We described a rare case in a non-HIV Greek female patient who had a tibial cryptococcal chronic osteomyelitis as she was on immunosuppressive treatment for rheumatoid arthritis. The most unusual finding was the coexistence of cryptococcal osteomyelitis with a subcutaneous lesion of the breast. She had a rigorous improvement in the inflammatory signs on final examination as an outpatient in her tibia and the breast.

## Data Availability

All available.

## Abbreviations

HIV: Human immunodeficiency virus
CT: Computed tomography
CRP: C-reactive protein
ESR: Erythrocyte sedimentation rate
PCT: Procalcitonin
mg: Milligram
kg: Kilogram
cm: Centimeter
ng: Nanogram
μL: Microliter
MRI: Magnetic resonance imaging
STIR: Short tau inversion recovery
i.v.: Intravascular
ps: Present case
